# Multidimensional High-Resolution Magic Angle Spinning and Solution-State NMR Characterization of ^13^C-labeled Plant Metabolites and Lignocellulose

**DOI:** 10.1038/srep11848

**Published:** 2015-07-06

**Authors:** Tetsuya Mori, Yuuri Tsuboi, Nobuhiro Ishida, Nobuyuki Nishikubo, Taku Demura, Jun Kikuchi

**Affiliations:** 1 Graduate School of Bioagricultural Sciences, Nagoya University, 1 Furo-cho, Chikusa-ku, Nagoya 464-0810, Japan; 2 Biotechnology Laboratory, Toyota Central R&D Labs, Inc., 41-1, Nagakute 480-1192, Japan; 3 RIKEN Center for Sustainable Resource Science, 1-7-22 Suehiro-cho, Tsurumi-ku, Yokohama 230-0045, Japan; 4 Biomass Engineering Program, RIKEN Research Cluster for Innovation, 2-1 Hirosawa, Wako 351-0198, Japan; 5 Graduate School of Medical Life Science, Yokohama City University, 1-7-29 Suehiro-cho, Tsurumi-ku, Yokohama 230-0045, Japan

## Abstract

Lignocellulose, which includes mainly cellulose, hemicellulose, and lignin, is a potential resource for the production of chemicals and for other applications. For effective production of materials derived from biomass, it is important to characterize the metabolites and polymeric components of the biomass. Nuclear magnetic resonance (NMR) spectroscopy has been used to identify biomass components; however, the NMR spectra of metabolites and lignocellulose components are ambiguously assigned in many cases due to overlapping chemical shift peaks. Using our ^13^C-labeling technique in higher plants such as poplar samples, we demonstrated that overlapping peaks could be resolved by three-dimensional NMR experiments to more accurately assign chemical shifts compared with two-dimensional NMR measurements. Metabolites of the ^13^C-poplar were measured by high-resolution magic angle spinning NMR spectroscopy, which allows sample analysis without solvent extraction, while lignocellulose components of the ^13^C-poplar dissolved in dimethylsulfoxide/pyridine solvent were analyzed by solution-state NMR techniques. Using these methods, we were able to unambiguously assign chemical shifts of small and macromolecular components in ^13^C-poplar samples. Furthermore, using samples of less than 5 mg, we could differentiate between two kinds of genes that were overexpressed in poplar samples, which produced clearly modified plant cell wall components.

An expanding global population in competition for decreasing natural resources, including crude oil, food, fertilizers, and metals, poses a serious challenge to human existence, and the time has come for a radical overhaul of alternative resources and their sustainable use. Increasing crop production without an excessive supply of fertilizers and water resources^1^ has resulted in an increase in inedible plant biomass, which is believed to be a renewable energy supply and an alternative raw material^2^. Plant biomass is mainly composed of cellulose, hemicellulose, and lignin, which accumulate in the cell wall^3^. Polymeric components of plant metabolites are synthesized from low-molecular-weight metabolites^4^. Monitoring these polymeric components is useful in biorefineries, where biomass is converted into useful materials. Thus, developing technology to evaluate plant biomass components is valuable in order to identify plants with mutations capable of improving biomass production^5^,^6^. Current methods include X-ray diffraction^7^, infrared spectroscopy^8^, microscopy^9^, mass spectrometry^10^, and nuclear magnetic resonance (NMR) spectroscopy^11^.

NMR has proven to be one of the more useful methodologies for analyzing biomolecular mixtures of metabolites and polymeric components^12^,^13^. ^1^H-NMR is a non-destructive and cost-effective method^14^, which also allows for quantitative evaluation of dynamic balance changes in the water-soluble metabolites included in various plants^15^. While ordinary metabolic profiling based on solution NMR is commonly used for samples extracted from tissues, the high-resolution magic angle spinning (HR-MAS) technique has been used to profile metabolites in intact organs^16^,^17^. HR-MAS analysis using intact tissues of plant samples has been reported for red algae^18^, wheat flour^19^, carrot^20^, and Arabidopsis^21^ and these results indicate that the HR-MAS technique can be applied to the detection of metabolites in other plants^22^,^23^.

Various solid and solution-state NMR techniques have been used to detect polymeric components of plant. The solid-state NMR technique is useful for analyzing macromolecules in intact plant samples, e.g., beech wood, pine^24^, and wheat straw^25^. It is, however, difficult to assign each NMR resonance using solid-state methods because of its low resolution^26^. On the other hand, solution-state NMR techniques have been used for analyzing plant samples that were ball-milled and then dissolved in dimethylsulfoxide (DMSO) solvent^27^, and these samples included many polymeric components. In a recent study, various polymeric components were characterized by solution-state two-dimensional (2D) NMR studies of ball-milled biomass gels in DMSO/pyridine^28^ and pyridinium chloride/DMSO^26^ systems. These methods are advantageous in acquiring detailed information on biomass because high-resolution NMR signals are provided by solution-state 2D NMR experiments, such as ^1^H-^13^C heteronuclear single-quantum coherence (HSQC)^29^,^30^,^31^,^32^. Although this method can lead to partial assignment of chemical shifts in the spectra of polymeric components, it is difficult to completely assign the spectra due to degeneracy resulting in overlapping chemical shift peaks. Conversely, separation of chemical shifts can be accomplished more readily with three-dimensional (3D) pulse sequences employing ^13^C-^13^C correlations^33^,^34^,^35^. However, because of the low natural abundance of ^13^C (1.1%), samples must be enriched by ^13^C stable isotope labeling^36^,^37^,^38^,^39^,^40^,^41^, which allows more reliable signal assignment using 3D pulse sequences^33^,^34^,^35^. For example, using the ^13^C labeling method, the uptake of [^13^C_6_]glucose via roots and the assimilation of ^13^CO_2_ into higher plants can be monitored^42^. It is deduced that the NMR analysis of biomass using 13C labeling technique provides advantages. For example, it can investigate biomass components with molecular motion^43^, and its experimental time for the 2D NMR profiling can be shorten comparing to that using non-labeled samples^44^.

In this report, we describe the results of our multidimensional NMR analysis of metabolites in plant samples prepared with ^13^C labeling. We selected poplar for our plant samples, because poplar is one of the main sources of plant biomass, and is easily transformed^45^. To analyze low-molecular-weight metabolites in the samples, an intact poplar sample was measured by HR-MAS 2D ^1^H-^13^C HSQC^46^ and 3D HCCH correlation spectroscopy (COSY)^47^,^48^, which does not require a solvent extraction process. To analyze polymeric components in the samples, substances that were ball-milled and then dissolved in DMSO/pyridine solvent were measured by solution-state 2D HSQC and 3D HCCH total correlation spectroscopy (TOCSY)^49^ NMR. More NMR signals of plant metabolites could be assigned because of the high resolution provided by the combination of ^13^C-labeling and 3D NMR techniques. To demonstrate an application of our methodology, we then compared plant metabolites produced in two kinds of genes overexpressed in poplar using samples of less than 5 mg. Concept of this study is summarized in Fig. 1.

## Results

### Analysis of ^13^C-poplar metabolites without extraction.

**Figure 2:**
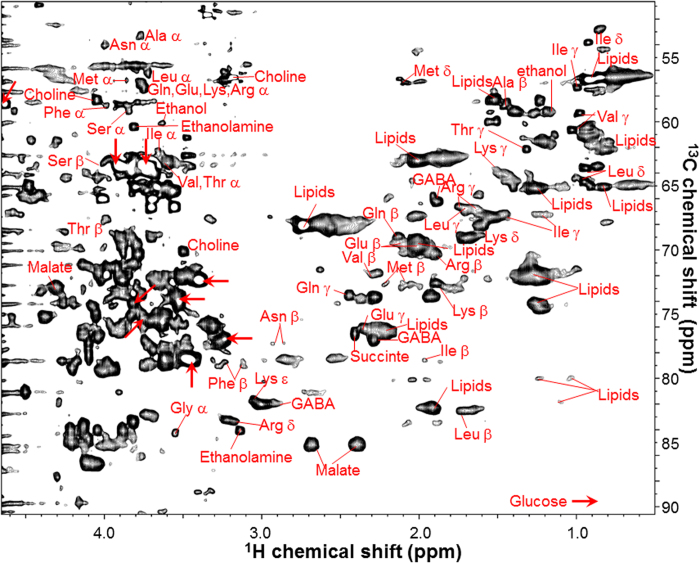
HR-MAS ^1^H-^13^C HSQC spectrum of ^13^C-poplar, measured without sample extraction. Peaks in the spectrum corresponding to metabolites were assigned by 3D HCCH-COSY experiments and matched by standard metabolites and SpinAssign. Ala, Alanine; Glu, Glutamic acid; Phe, Phenylalanine; Gly, Glycine; Ile, Isoleucine; Lys, Lysine; Leu, Leucine; Met, Methionine; Asn, Asparagine; Gln, Glutamine; Arg, Arginine; Ser, Serine; Thr, Threonine; Val, Valine; GABA, γ-amino butyric acid. The arrows show Glucose signals.

**Figure 3:**
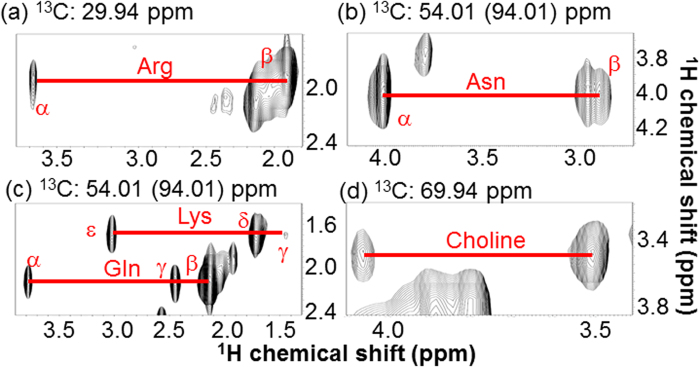
HR-MAS 3D HCCH-COSY spectrum of ^13^C-poplar, measured without sample extraction. 2D ^1^H-^1^H planes at (**a**) 29.94, (**b**) 54.01 (folded spectrum; 94.01), (**c**) 54.01 (94.01), and (**d**) 69.94 ppm of ^13^C axis of the 3D ^1^H-^1^H-^13^C spectrum are shown. Red lines connect the ^1^H-^13^C-^13^C-^1^H cross peaks of the assigned metabolites. Asn, Asparagine; Arg, Arginine; Lys, Lysine; Gln, Glutamine.

We prepared a poplar sapling with ^13^C- plant biomass for multidimensional NMR measurements. First, an intact poplar was measured by HR-MAS. Figure 2 shows the aliphatic region in the HR-MAS 2D ^1^H-^13^C-HSQC spectrum of the ^13^C-poplar sample. Although these ^1^H-^13^C signals were slightly broadened in the ^1^H dimension by residual ^1^H-^1^H dipolar interactions, chemical shift dispersion could be adequately resolved in the intact tissues. In the HR-MAS ^1^H-^13^C-HSQC spectrum of the sample, various amino acids, ethanol, malate, choline, ethanolamine, and glucose were assigned by matches using the ^1^H and ^13^C chemical shift database SpinAssign^50^,^51^. The SpinAssign database, which is a database of standards dissolved in potassium phosphate buffer, is useful to analyze the HR-MAS HSQC spectrum of intact ^13^C-poplar because of similarities in chemical shifts when comparing the potassium phosphate buffer with an intracellular environment. During the process of matching chemical shift data for each signal, we frequently observed multiple candidate metabolites in the database, indicating that the candidates may include false-positive matches. For example, three signals, (3.757 of ^1^H, 57.160 ppm of ^13^C), (2.128, 68.947), and (2.442, 73.535), in the spectrum were matched to the chemical shifts of glutamine α, β, and γ, respectively, in a recent SpinAssign database (unpublished data). However, by integrating the matched signals, the individual signals were initially matched to 10, 3, and 2 candidate metabolites, respectively, in the results of the SpinAssign search. The candidate metabolites did include the chemical shifts of glutamine α, β, and γ, i.e., true-positive matches. The occurrence of false-positives matches was discussed in a previous study^33^. ^13^C-lipid, which is unregistered in SpinAssign, was measured by HR-MAS, and then matched with chemical shifts for ^13^C-poplar. To assign the 2D ^1^H-^13^C-HSQC signals on the basis of the information from ^1^H-^13^C-^13^C-^1^H correlations, the 3D HCCH-COSY spectrum (Fig. 3) of the sample was recorded. Figure 3 shows 4 ^1^H-^1^H planes slicing the 3D ^1^H-^1^H-^13^C spectrum of the intact tissue sample at different points along the ^13^C axis. Various amino acids, ethanol, malate, choline, ethanolamine, and glucose were assigned on the basis of ^1^H-^13^C-^13^C-^1^H correlations by 3D HCCH-COSY to avoid false-positive matches.

### Polysaccharide analysis of plant metabolites

**Figure 4:**
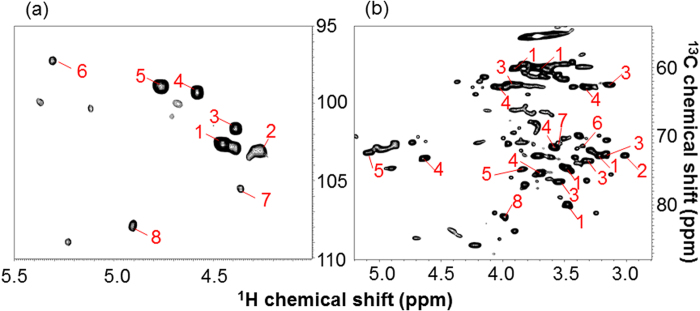
Solution-state ^1^H-^13^C HSQC spectrum of ^13^C-poplar extracted in DMSO/pyridine. Peaks were assigned by 3D HCCH-TOCSY experiments and matched on the basis of the results reported by Kim *et al.* (2010). Peak numbers in the figure correspond with those listed in Table 1. (a) Anomeric region. (**b**) Aliphatic region.

**Figure 5:**
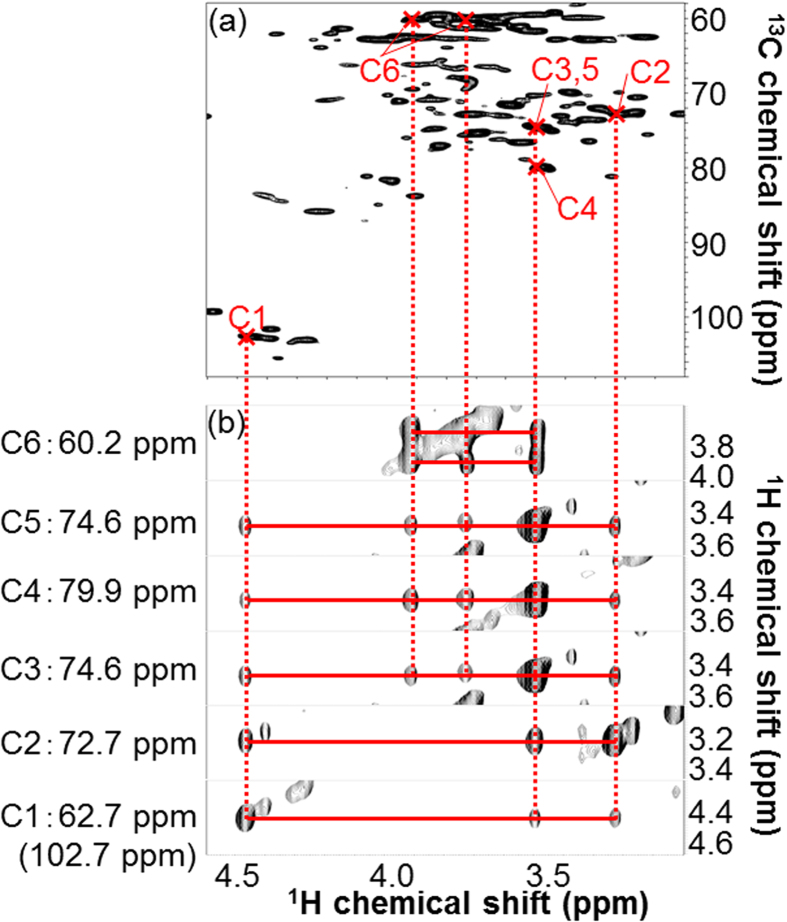
Analysis of (1, 4)-β-d-Glc*p* using solution-state 2D HSQC and 3D HCCH-TOCSY spectra of ^13^C-poplar extracted in DMSO/pyridine. (**a**) 2D ^1^H-^13^C HSQC spectrum. Crossed marks show C1-6 signals of (1, 4)-β-d-Glc*p* assigned by 3D HCCH-TOCSY. (**b**) 2D ^1^H-^1^H planes at 60.2, 74.6, 79.9, 74.6, 72.7, and 62.7 (folded spectrum; 102.7) ppm of ^13^C, which correspond to the C6, C5, C4, C3, C2, and C1 of (1, 4)-β-d-Glc*p*, slicing the 3D ^1^H-^1^H-^13^C spectrum. Red transverse lines connect ^1^H-^13^C-^13^C-^1^H cross peaks and vertical dashed lines connect corresponding signals between 3D and 2D spectra.

To detect polysaccharides, we measured the sample dissolved in DMSO/pyridine by 2D ^1^H-^13^C-HSQC (DMSO/pyridine system). Figure 4 shows the anomeric and aliphatic regions of the HSQC spectrum. Peaks detected in the anomeric region were matched [Fig. 4(a)] on the basis of previously reported results^28^, and then peaks detected in the anomeric and aliphatic regions were assigned (Figs 4 and 5) on the basis of the 3D HCCH-TOCSY spectrum of the sample. The peak numbers in Fig. 4 correspond with those in Table 1. Figure 5(b) shows 6 ^1^H-^1^H planes slicing the 3D ^1^H-^1^H-^13^C spectrum along the ^13^C axis of the C1-C6 chemical shifts of (1, 4)-β-d-glucopyranoside [i.e., (1, 4)-β-d-Glc*p*] derived from cellulose, glucan derived from xyloglucan, and/or glucan derived from glucomannan. Using the 3D HCCH-TOCSY, ^1^H-^13^C-^13^C-^1^H correlations through multiple bonds are detected (i.e., multiple adjacent correlations). Based on the connections of C1-C6 in the 3D HCCH-TOCSY, (1, 4)-β-d-Glc*p* was completely assigned [Fig. 5(a)]. Also, 2-O-acetyl-β-d-xylopyranoside (2-O-Ac-β-d-Xyl*p*) derived from xylan, which has O-acetyl groups attached at the C2 position, was completely assigned (Table 1). Although one polysaccharide was completely assigned using carbon-carbon correlations like (1, 4)-β-d-Glc*p* and 2-O-Ac-β-d-Xyl*p*, the signals did not correspond with those of standards and references. This polysaccharide was designated as ‘unknown polysaccharide X’. Partial assignments were also completed for unknown polysaccharide G; 2-O-acetyl-β-d-mannopyranoside 2-O-Ac-Man*p* derived from mannan, to which O-acetyl groups were attached at the C2 position; 4-*O*-methyl-α-d-glucuronic acid (4-*O*-Me-GlcA) derived from glucuronic acid, to which O-methyl groups and xylan residue were attached at C4 and C1, respectively; β-d-galactopyranoside (β-d-Gal*p*) derived from galactan; and α-l-arabinofuranoside (α-l-Ara*f*) derived from arabinan or arabinose, to which a xylan residue was attached at C1.

We analyzed the samples to detect polysaccharides using HR-MAS and solid-state NMR techniques. In the HR-MAS analysis, the HSQC spectrum of a cell-wall-rich sample prepared from ^13^C-poplar was compared with those three commercial pectin standards. The NMR signals of the sample matched to 18 out of 20 apple pectin signals, 27 out of 31 rhamnogalacturonan-I signals, and 27 out of 35 arabinogalactan signals (Figure S1). In the solid-state NMR analysis, 2D ^13^C–^13^C refocused Incredible Natural Abundance DoublE QUAntum Transfer Experiment (INADEQUATE) NMR spectra^52^ of ^13^C-poplar were obtained using two different τ delay times to detect optimal signal intensities of polysaccharides [Figure S2(b)] and aliphatic compounds [Figure S2(c)]. The NMR signals were characterized using chemical shift data for cellulose Iβ^40^, amorphous cellulose^53^, lipids^54^,^55^, and amino acids^50^,^56^. The MAS-*J*-HMQC^57^ signals of ^13^C-poplar were also used to characterize *J*-based ^13^C-^1^H cross peaks of the signals assigned using the characterization [Figure S2(a)].

### Application to comparison of mutants [overexpression of VASCULAR-RELATED NAC-DOMAIN 6 (VND6) and VND7].

**Figure 6:**
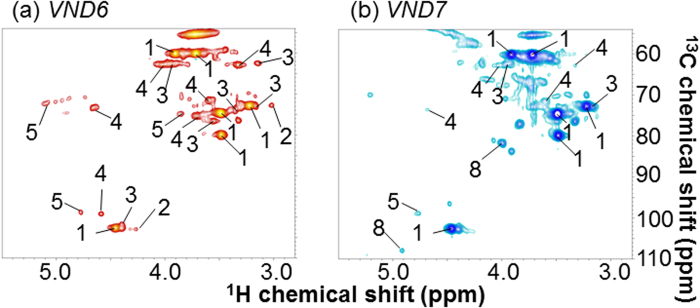
Solution-state ^1^H-^13^C HSQC spectra of two transgenic ^13^C-poplar samples of less than 5 mg each dissolved in DMSO/pyridine, following subtraction of wild-type spectrum. Peak numbers correspond with those in Table 1. (a) *VND6*. (**b**) *VND7*. Blue signals are positive, and red signals are negative.

Based on the assignment of the signals of both metabolites and polymeric components, we applied these NMR studies to mutant characterization. We prepared two transgenic poplars by overexpression of *VND6* and *VND7* genes^58^. *VND6* and *VND7* are reported to be master regulator genes involved in the formation of vessels during the development of the metaxylem and protoxylem, respectively, because overexpression of these genes can induce trans-differentiation of various cells into metaxylem- and protoxylem-like vessel elements, respectively, in both *Arabidopsis* and poplar^58^. *VND* genes are anticipated to play an important role in the study of biomass production^59^. HR-MAS analyses of these two mutant samples revealed a dramatic difference in their metabolic profiles, based on their 2D NMR spectra (Figure S3). Furthermore, polymeric components of the two transgenic poplars were compared. Figure 6 shows spectra of the anomeric and aliphatic regions in the HSQC experiments of *VND6* [Fig. 6(a)] and *VND7* [Fig. 6(b)], following subtraction of the wild-type spectrum. (1, 4)-β-d-Glc*p*, unknown polysaccharides G and X, 2-O-Ac-β-d-Xyl*p*, and 2-O-Ac-Man*p* detected in the *VND6* experiment were less evident than those found in the wild-type samples. In contrast, (1, 4)-β-d-Glc*p*, unknown polysaccharide X, 2-O-Ac-β-d-Xyl*p*, 2-O-Ac-Man*p*, and α-l-Ara*f* detected in the *VND7* experiment were more evident than those found in the wild-type experiment.

## Discussion

3D NMR measurements combined with ^13^C isotope labeling techniques are an effective approach for identifying polymeric components and metabolites. Using 3D NMR measurements such as HCCH-COSY and HCCH-TOCSY improves peak resolution compared with 2D NMR measurements, and thus, complete assignment of polymeric components and metabolites is anticipated. However, the low natural abundance of ^13^C produces low signal intensity that inhibits detection of ^13^C-^13^C correlations in 3D NMR measurements. By enhancing the signals by ^13^C isotope labeling, we were able to completely assign polymeric components and metabolites (e.g., glucopyranose) within polysaccharides.

A number of peaks from the HR-MAS 2D ^1^H-^13^C-HSQC spectrum of ^13^C-poplar were matched to particular metabolites using the SpinAssign database, including glutamine and arginine. However, we also encountered a problem with this approach, which may list matched metabolites with many false-positive candidates. Two solutions to the false-positive assignment have been proposed^33^: (1) to combine multiple 2D NMR experiments that are used to identify compounds in complex mixtures^60^,^61^ and (2) to introduce heteronuclear 3D NMR spectra for reducing the number of ambiguous assignments^34^.

Thus, by introducing 3D NMR measurements for reducing false-positive assignments, we were able to assign signals of metabolites such as arginine and asparagine in the 2D ^1^H-^13^C-HSQC spectrum on the basis of ^13^C-^13^C correlations observed in the 3D HCCH-COSY spectrum. However, ^1^H-^13^C-^13^C-^1^H correlations for saccharides could not be determined in the 3D HCCH-COSY except for glucose, because a substantial number of peaks associated with monosaccharides and oligosaccharides were detected in the sugar region (3.0–4.7 in the ^1^H dimension and 50–90 ppm in the ^13^C dimension) of the 2D spectrum (Fig. 2). Assignment of these peaks will likely require the development of new, higher resolution NMR methods.

Although the HR-MAS technique is capable of measuring intact samples, many metabolites were not detected using this approach. However, we were able to identify these metabolites by comparing the results of HR-MAS with those obtained by solution-state NMR techniques. By comparing the HR-MAS spectrum from our intact sample (Fig. 2) with the solution-state NMR spectrum from the sample extracted by potassium phosphate buffer (Figure S4), we observed the localization of metabolite signals that were undetected by HR-MAS, but detected by solution-state NMR. For example, asparagine, citrate, and unassigned metabolites were detected in the solution-state NMR spectrum (Figure S4), but undetected in the HR-MAS spectrum (Fig. 2), indicating that their metabolites were localized in a particular organelle. In the HR-MAS technique using an intact sample, this phenomenon may be attributed to the limited motion reflected by localization in a particular organelle such as the mitochondrial membrane^17^,^62^,^63^,^64^,^65^. Conversely, since solution-state NMR requires the extraction of components, their composition is varied and depends on the solvents used for extraction. However, this limitation is not present with HR-MAS because it works with intact samples. This allows us to observe lipid chains, ethanol, leucine, isoleucine, and other unassigned aliphatic side chains, as shown in Fig. 2. Because solution-state NMR avoids the localization problem, characterization is significantly improved by combining solution-state NMR and HR-MAS techniques.

In the solution-state 2D ^1^H-^13^C-HSQC NMR spectrum of ^13^C-poplar that was ball-milled and then dissolved in DMSO/pyridine solvent, we could detect a substantial number of peaks corresponding to polymeric components (e.g., polysaccharides). The signals detected in the polysaccharide anomeric region of the ^1^H-^13^C-HSQC spectrum were assigned on the basis of the results of a previous study^28^, in which the anomeric carbons of polysaccharides were identified. However, we were only able to identify anomeric carbons of polysaccharides by this approach; therefore, to identify other anomeric carbon signals, we combined 3D NMR experiments with 2D experiments, similar to the HR-MAS technique. By combining 2D and 3D measurements, we assigned additional polysaccharide peaks, except for the anomeric regions of the 2D ^1^H-^13^C-HSQC spectrum, on the basis of ^13^C-^13^C correlations by 3D NMR. Using this method, we completely assigned several polysaccharides in our sample. For example, using the 3D HCCH-TOCSY, ^1^H-^13^C-^13^C-^1^H correlations from C1 to C6 of (1, 4)-β-d-Glc*p* were confirmed (Fig. 5). However, some polysaccharides were only partially assigned due to the low intensities of the 3D NMR signals of these polymeric components. These results indicate that detection of small amounts of polymeric components will require different methods to improve signal sensitivity. The assignment of acetylated hemicelluloses cause the migration of acetyl groups during the preparation of the NMR sample^66^.

We detected pectin-like polysaccharide components in the HR-MAS NMR spectrum of the cell-wall-rich sample of ^13^C-poplar (Figure S1). Therefore, this method is useful to detect gel-like, faster molecular motion polysaccharides such as pectin. However, cellulose and hemicellulose were not detected using this approach because of the slower molecular motion of these polysaccharides. Thus, we analyzed the macromolecules of ^13^C-poplar using solid-state NMR (Figure S2). Although we could detect polysaccharides, lipids, and protein-derived materials using two different τ delay times in the refocused INADEQUATE method, it was difficult to make detailed assignments for these compounds. Therefore, the combined use of higher resolution solution-state NMR with solid-state NMR might be a complementary approach to characterize cell-wall components.

We applied the assignments of polysaccharides to our analysis of the transgenic *VND6* and *VND7* strains of ^13^C-poplar using samples of less than 5 mg, in which protoxylem and metaxylem vessel formation were introduced, respectively^58^. Metaxylem and protoxylem are formed respectively at the early and late stages of primary xylem formation. In the overexpression measurements of *VND6* and *VND7*, polymeric components were anticipated to be observed at both late (i.e., protoxylem) and early (i.e., metaxylem) stages. Our poplar samples were grown for short durations, i.e., early stage, and thus, polymeric components of *VND7* were longer than those in the wild-type, and in contrast, *VND6* components were shorter than those in the wild-type (Fig. 6). The results of the HR-MAS analyses of *VND6* and *VND7* of ^13^C-poplar supported the polymeric components data (Figure S3). Sugars based on polysaccharides of *VND7* were shorter than those in the wild-type, because many sugars would have been used to synthesize polysaccharides. In contrast, the sugars of *VND6* were longer than those in the wild-type, because fewer sugars would have been used to synthesize polysaccharides. These data indicate that polymeric component analysis by the DMSO/pyridine system is useful for comparing transgenic organisms. Namely, the ^13^C-labeling technique allows reduction of the required sample amounts, e.g., less than 5 mg in our case, to approximately 10-fold lower than the ordinary case. This study may, therefore, provide valuable and detailed information relating to the improvement of biomass production.

## Method

### Preparation of poplar samples

All experiments were conducted using poplar hybrid aspen (*Populus tremula* L. × *Populus tremuloides* Michx.), grown in a plant incubation room (16-h day/8-h night, 60 μmol·m^−2^·s^−1^ light intensity, at 23 °C). Transformation and regeneration of the poplar were carried out as described previously^58^,^67^. For shoot amplification, Murashige and Skoog (MS) medium (Sigma-Aldrich, St. Louis, MO), which contains indole-3-butyric acid and 6-benzylaminopurine, was placed on a sterilized plate with stalks of cut poplar stems. After approximately 30 days of rooting, the shoots were transferred to plant culture test tubes (IWAKI, Chiba, Japan) containing MS medium. After approximately 30 additional days, the rooted poplars were transferred to a container containing MS medium for plant culture (Combiness, Nazareth, Belgium). Stable isotope labeling of poplars using the above growing system was conducted using previously described methods^38^,^42^. The poplars were grown in the plant culture until they reached a height of approximately 10 cm, i.e., 35 days.

### Extraction and solution NMR of poplar

The lyophilized ^13^C-labeled poplar (^13^C-poplar) was crushed and extracted using previously described methods^68^. Briefly, aqueous buffer (100 mM potassium phosphate, pH 7.0) was used for extraction. Solution NMR experiments were performed using a DRU-700 spectrometer (Bruker Biospin, Billerica, MA, USA) equipped with a Z-axis cryogenically cooled probe operating at 25 °C. For 2D ^1^H-^13^C-HSQC analysis, a total of 128 complex f1 (^13^C) and 1,024 complex f2 (^1^H) points were recorded using 80 scans per f1 increment. The spectral widths and offset frequencies were 7,042 Hz (40 ppm) and 9,328 Hz (13.3 ppm) for f1 and f2, respectively. The chemical shifts were referenced to the methyl group of the sodium 2,2-dimethyl-2-silapentane-5-sulfonate (DSS) internal standard (0 ppm of ^1^H and 0 ppm of ^13^C).

### Preparation of insoluble cell-wall-rich sample from poplar

The extracted residue sample of ^13^C-poplar was prepared using previously described methods^34^. Briefly, chloroform, methanol, and sodium dodecyl sulfate were used to remove low-molecular-weight metabolites, lipids, and proteins from the poplar sample.

### HR-MAS measurements of poplar sample, insoluble cell-wall-rich poplar sample, lipids, and pectins

HR-MAS measurements of the samples and standards without extraction were conducted using DRX-400 and DRX-500 spectrometers (Bruker Biospin, Billerica, MA, USA) equipped with Z-axis high-resolution magic angle spinning probes. The measurement temperature was maintained at 25 °C. The MAS rotational speed was regulated at a constant 4,000, 10,000, and 6,000 Hz for the 3D analysis of the poplar sample, the cell-wall-rich sample, and others, respectively. For 2D ^1^H-^13^C-HSQC measurements, the DRX-500 spectrometer was used and a total of 160 complex f1 (^13^C) and 1,024 complex f2 (^1^H) points were recorded using 56 scans per f1 increment. The spectral widths and offset frequencies were 5,031 Hz (40 ppm) and 6,667 Hz (13.3 ppm) for f1 and f2, respectively. For 3D HCCH-COSY measurements, the DRX-400 spectrometer was used and a total of 120 complex f1 (^1^H), 56 complex f2 (^13^C), and 1,024 complex f3 (^1^H) points were recorded using 24 scans per f1 and f2 increments. The spectral widths and offset frequencies were 4,802 Hz (12.0 ppm), 4,025 Hz (40 ppm), and 5,593 Hz (14.0 ppm) for f1, f2, and f3, respectively. The water phase, including the sample in the rotor, was retained under MAS conditions. The chemical shifts were referenced to the methyl group of the DSS internal standard.

### Solid NMR of poplar

Freeze-dried ^13^C poplar was inserted into 4-mm ø ZrO_2_ rotor. Solid NMR experiments were performed using a DRX-400 spectrometer (Bruker Biospin, Billerica, MA, USA) equipped with a 4-mm MAS triple resonance probe. The MAS rotational speed was regulated at a constant 13,500 Hz. For MAS-*J*-HMQC, 72 complex f1 (^1^H) and 768 complex f2 (^13^C) points were recorded using 160 scans per f1 increment. The spectral widths and offset frequencies were 11,043 Hz (27 ppm) and 25,253 Hz (250 ppm) for f1 and f2, respectively. The cross-polarization contact time was set to 3.0 ms. For refocused INADEQUATE, 96 complex f1 and 768 complex f2 points were recorded using 896 and 1,200 scans per f1 increment with the τ delay set to 3.4 and 6.0 ms. The spectral widths and offset frequencies were 48,077 Hz (480 ppm) and 24,038 Hz (240 ppm) for f1 and f2, respectively. The cross-polarization contact time was set to 2.0 ms.

### DMSO-d_6_/pyridine-d_5_ system

Freeze-dried ^13^C poplar was crushed in the same way as previously described^68^. The crushed sample was ball-milled with a FRITSCH pulversette P5 vibratory ball mill (FRITSCH, Idar-Oberstein, Germany) vibrating at 400 rpm using zirconium dioxide (ZrO_2_) vessels (50 mL) containing ZrO_2_ ball bearings (5 × 5 mm) for 12 h (in cycles comprising 10-min grinding/10-min interval). The milled sample was extracted with ethanol (shaking, 50 °C, 5 min, 3 times) and distilled water (shaking, 50 °C, 5 min, 3 times). The sample was dissolved in DMSO-*d*_6_/pyridine-*d*_5_ (4:1) (Cambridge Isotope Laboratories, Andover, MA), shaken at 50 °C for 30 min, and centrifuged at 20,000 *g* for 5 min. Solution NMR experiments were performed on the soluble matter from the sample using the DRU-700 spectrometer (Bruker Biospin, Billerica, MA, USA) equipped with a Z-axis cryogenically cooled probe operating at 45 °C. For 2D ^1^H-^13^C-HSQC measurements to assign polymeric components combined with 3D HCCH-COSY, a total of 512 complex f1 (^13^C) and 1,024 complex f2 (^1^H) points were recorded using 16 scans per f1 increment. The spectral widths and offset frequencies were 26,410 Hz (150 ppm) and 9,328 Hz (13.3 ppm) for f1 and f2, respectively. For 3D HCCH-TOCSY measurements, a total of 128 complex f1 (^1^H), 64 complex f2 (^13^C), and 1,024 complex f3 (^1^H) points were recorded using 24 scans per f1 and f2 increments. The spectral widths and offset frequencies were 7,002 Hz (10.0 ppm), 7,042 Hz (40 ppm), and 9,803 Hz (14.0 ppm) for f1, f2, and f3, respectively. The mixing time was set to 14 ms. For 2D ^1^H-^13^C-HSQC measurements of wild-type and two transgenic ^13^C-poplars, a total of 256 complex f1 (^13^C) and 1,024 complex f2 (^1^H) points were recorded using 16 scans per f1 increment. The spectral widths and offset frequencies were 26,410 Hz (150 ppm) and 9,328 Hz (13.3 ppm) for f1 and f2, respectively. The chemical shifts were referenced to a DMSO internal standard (2.49 ppm for ^1^H and 39.5 ppm for ^13^C).

### Data processing

Each NMR spectrum was processed using the NMRPipe software^69^ with appropriate window functions, zero-filling, linear predictions, and polynomial baseline corrections. Metabolites were assigned using a recent NMR chemical database^50^ (unpublished data), while polysaccharides were assigned, in part, on the basis of previously reported results^28^.

## Additional Information

**How to cite this article**: Mori, T. *et al.* Multidimensional High-Resolution Magic Angle Spinning and Solution-State NMR Characterization of ^13^C-labeled Plant Metabolites and Lignocellulose. *Sci. Rep.* **5**, 11848; doi: 10.1038/srep11848 (2015).

## Supplementary Material

Supplementary Information

## Figures and Tables

**Figure 1:**
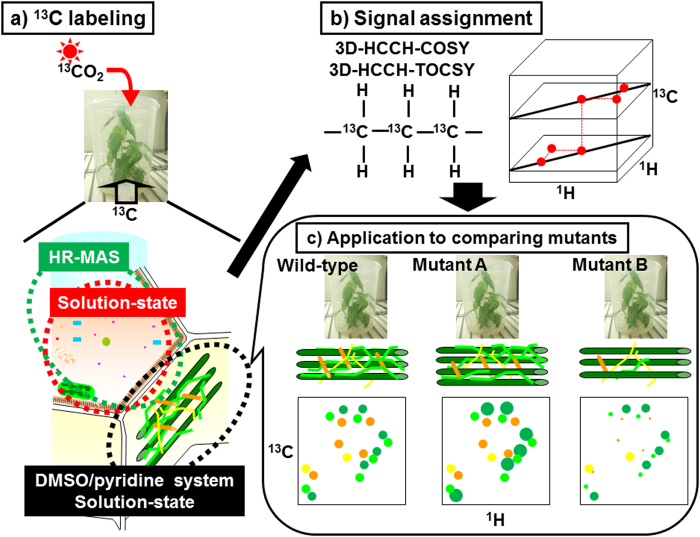
Diagrammatic illustration of our method for characterization of ^13^C-labeled plant metabolites and lignocellulose. (**a**) Intact sample and DMSO/pyridine extraction of ^13^C-labeled poplar were used for HR-MAS and solution-state NMR, respectively. (**b**) Signal assignments of metabolites and lignocellulose components could be achieved by 3D HCCH-COSY and HCCH-TOCSY. (**c**) Based on signal assignments of lignocellulose components, application to two transgenic ^13^C-poplar samples were compared with wild-type poplar.

**Table 1:** 1H and ^13^C chemical shift assignments for polysaccharide components from ^13^C-poplar, based on a combination of the 3D HCCH-TOCSY experiment and the results reported by Kim *et al*. (2010).

No	Saccharide	Chemical shift (ppm)					
		C1/H1	C2/H2	C3/H3	C4/H4	C5/H5	C6/H6
**1**	(1, 4)-β-D-Glc*p*	102.6/4.45	74.6/3.48	74.6/3/48	79.9/3.48	74.6/3.48	60.1/3.90, 60.1/3.72
**2**	Unknown polysaccharide G	103.1/4.27	72.8/3.01	ND	ND	ND	ND
**3**	Unknown polysaccharide X	101.6/4.39	72.6/3.17	73.6/3.34	76.6/3.55	62.5/3.93, 62.9/3.14	
**4**	2-O-Ac-β-D-Xyl*p*	99.3/4.58	73.2/4.64	71.6/3.59	75.4/3.67	62.9/4.02, 62.9/3.32	
**5**	2-O-Ac-Man*p*	98.9/4.76	72.4/5.10	74.8/3.84	ND	ND	ND
**6**	4-O-MeGlcA	97.2/5.31	71.4/3.35	ND	ND	ND	ND
**7**	(1, 4)-β-D-Gal*p*	105.5/4.36	71.0/3.55	ND	ND	ND	ND
**8**	α-L-Ara*f*	107.9/4.90	81.8/3.98	ND	ND	ND	

ND, not determined.
