# Supplementary material for: Multidimensional High-Resolution Magic Angle Spinning and Solution-State NMR Characterization of 13C-labeled Plant Metabolites and Lignocellulose
Source: Sci Rep. 2015 Jul 6;5:11848. doi: 10.1038/srep11848 (PMC4491710; doi:10.1038/srep11848)
Supplement: Supplementary Information [file srep11848-s1.doc]

**Additional information**

Multidimensional High-Resolution Magic Angle Spinning and Solution-State NMR Characterization of ^13^C-labeled Plant Metabolites and Lignocellulose

Tetsuya Mori^1,2,†^, Yuuri Tsuboi^3^, Nobuhiro Ishida^2^, Nobuyuki Nishikubo^3,‡^, Taku Demura^3.4^,

Jun Kikuchi^1,3,4,5^

^1^Graduate School of Bioagricultural Sciences, Nagoya University, 1 Furo-cho, Chikusa-ku, Nagoya 464-0810, Japan

^2^Biotechnology Laboratory, Toyota Central R&D Labs, Inc., 41-1, Nagakute 480-1192, Japan

^3^RIKEN Center for Sustainable Resource Science, 1-7-22 Suehiro-cho, Tsurumi-ku, Yokohama 230-0045, Japan

^4^Biomass Engineering Program, RIKEN Research Cluster for Innovation, 2-1 Hirosawa, Wako 351-0198, Japan

^5^Graduate School of Medical Life Science, Yokohama City University, 1-7-29 Suehiro-cho, Tsurumi-ku, Yokohama 230-0045, Japan

^†^Present address: RIKEN Center for Sustainable Resource Science, 1-7-22 Suehiro-cho, Tsurumi-ku, Yokohama 230-0045, Japan

^‡^Present address: Forest Technology Laboratories, Research & Development Division, Oji Paper Co., Ltd., 1-10-6 Shinonome, Koto-ku, Tokyo 135-8558, Japan

***Corresponding author:**

Jun Kikuchi

RIKEN Center for Sustainable Resource Science, 1-7-22 Suehiro-cho, Tsurumi-ku, Yokohama

230-0045, Japan

Tel: +81 45 503 9490; Fax: +81 45 503 9489;

Email: jun.kikuchi@riken.jp

**Supplementary Figure**

**
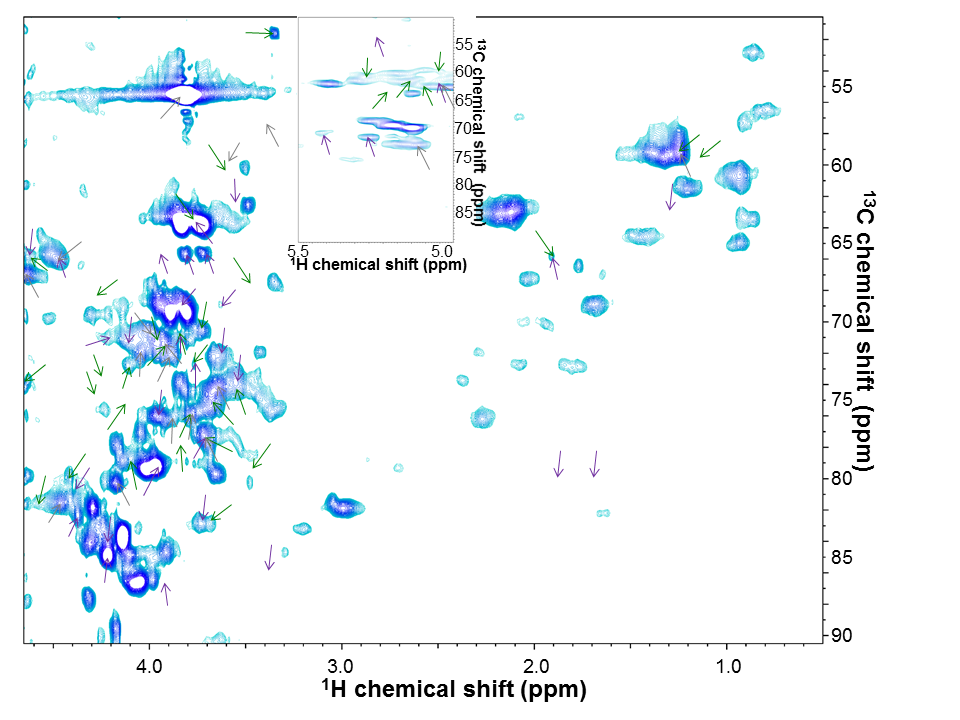
**

**Figure S1.** HR-MAS ^1^H-^13^C HSQC spectrum of insoluble cell-wall-rich sample of ^13^C-poplar. The arrows are highlighting signals derived from three different kinds of standard pectins (green; rhamnogalacturonan I, purple; arabinogalactan, and gray; apple pectin standards), although some signals were missing due to chemical shift changes or unknown reason.


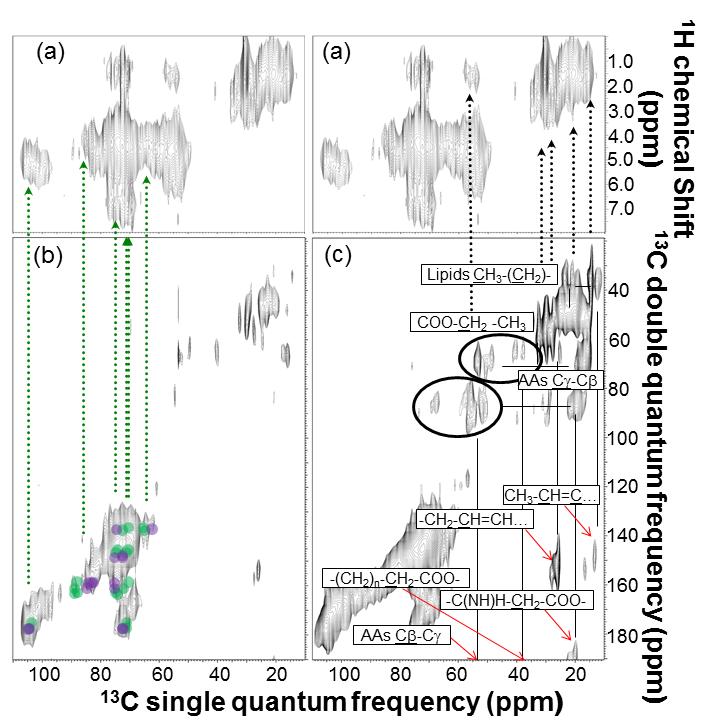
**Figure S2.** Solid-state NMR spectra of ^13^C-poplar. (a) MAS-*J*-HMQC. (b,c) Refocused INADEQUATE ; τ delay set to (b) 3.4 and (c) 6.0 ms. Green and purple circles represent chemical shift of cellulose Iβ and amorphous cellulose. AAs, amino acids.


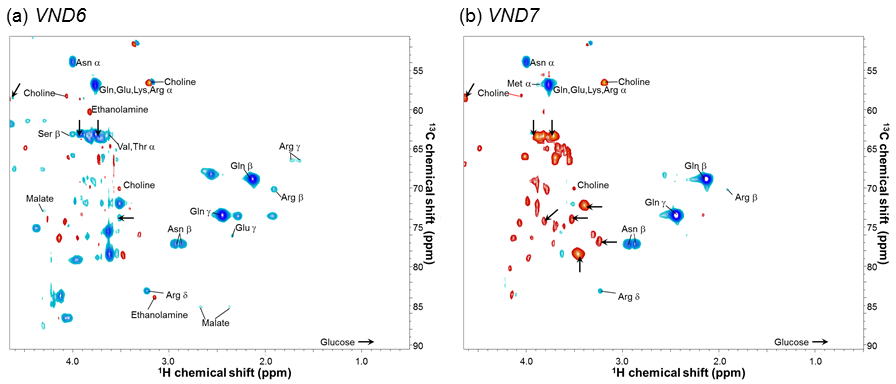


**Figure S3.** HR-MAS ^1^H-^13^C HSQC spectra of two transgenic ^13^C-poplar samples, after subtraction of wild-type spectrum. (a) *VND*6. (b) *VND7*. Blue signals, positive; red signals, negative. Glu, Glutamic acid; Met, Methionine; Asn, Asparagine; Gln, Glutamine; Arg, Arginine; Ser, Serine; Thr, Threonine; Val, Valine. The arrows are highlighted as Glucose signals.


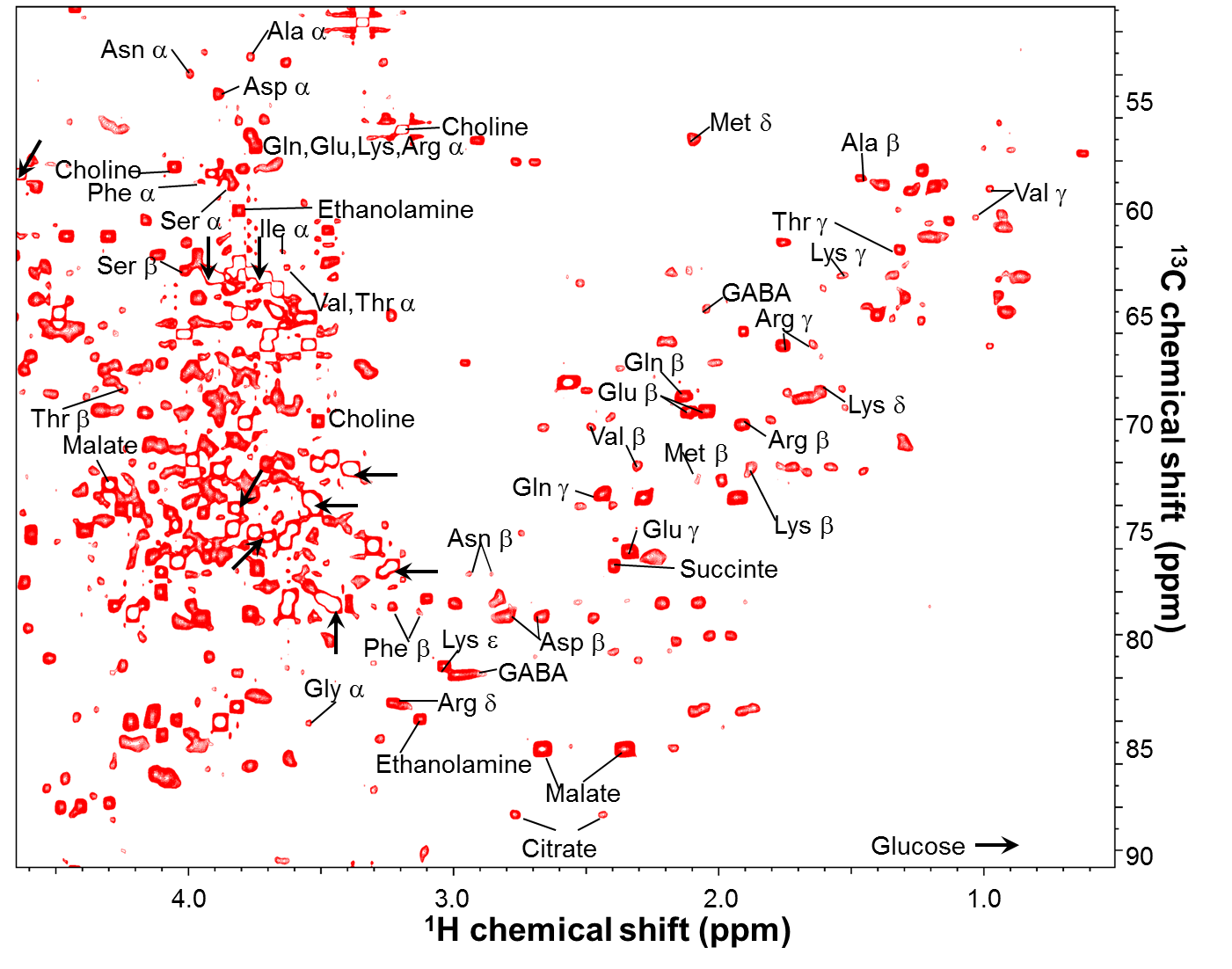


**Figure S4.** Solution-state ^1^H-^13^C HSQC spectrum of ^13^C-poplar extracted in 100 mM potassium phosphate buffer. Peaks in spectrum corresponding to metabolites were matched to specific compounds by SpinAssign, a ^1^H and ^13^C chemical shift database. Ala, Alanine; Asp, Aspartic acid; Glu, Glutamic acid; Phe, Phenylalanine; Gly, Glycine; Lys, Lysine; Met, Methionine; Asn, Asparagine; Gln, Glutamine; Arg, Arginine; Ser, Serine; Thr, Threonine; Val, Valine; GABA, γ-amino butyric acid. The arrows show Glucose signals.
